# Hydrate of Chloral

**Published:** 1874-05

**Authors:** E. M. Nolan

**Affiliations:** McDonough, GA.


### MY EXPERIENCE WITH
### HYDRATE OF CHLORAL.

BY E. M. NOLAN, M.D., McDONOUGH, GA.

The profession and people generally should be under lasting obligations to Liebreich, the discoverer of chloral. He has conferred upon them a blessing that can not be over estimated. In my humble opinion it is the finest hypnotic that we possess. It is very true that it does not act much as an anodyne, but as the discoverer in placing it before the profession did not intend it to be used as such, we should take it simply for what it is worth.

I have made quite an extensive trial of it, and do unhesitatingly affirm that it is the best of its class in use. And moreover, I believe the reason that it is not more popular with some physicians, is on account of their not using it judiciously and in the proper dose. One writer says that seventy grains of chloral is equal to half grain of morphia employed hypodermically. I have not found this to be so, but have seen as happy a soporific effect derived from twelve grains of hydrate of chloral, as from one-third grain of morphine hypodermically. I administer it in simple solution in water, and have never seen it cause vomiting or the least sickness of stomach. I have never seen complete anæsthesia or total loss of muscular power, from a dose of the drug. But have given it to a patient with hydrothorax when it produced a perfect hypnotic action with relief from the orthopnœa, and he would get out of bed and micturate or get a drink of water, all the time being perfectly unconscious; and the next morning he would say that he was very much refreshed, and that the rest procured from the chloral was delightful; and this from a dose of from ten to fifteen grains, in which dose I found it to act the best. I have tried it, though, in doses of thirty or forty grains. Given thus, the sleep was not so pleasant, and was disturbed. The patient appeared to be intoxicated, and when he would get up from his bed he would stagger across the room and be in great danger of falling over chairs, etc.

It has one great advantage over most of the other soporifics, and especially over opium and its preparations; it is not in the least degree constipating. With some it acts rather as a laxative. I have given it at night to patients, who were somewhat constipated, and next morning there would be a gentle but free evacuation from the bowels.

A few months ago a lady at her catamenial period requested relief from a very distressing and painful headache. I dissolved fifteen or twenty grains of chloral in very little water, and with the tip of one finger, rubbed it upon one of her temples until she could very sensibly feel the burning, and until the skin was reddened. The part rubbed was not larger than a silver dollar, but when I ceased the pain was entirely relieved and remained so. I have used it in this manner many times since, in ordinary nervous headache, and have never been disappointed, save one time, and then the patient was partially relieved. I sometimes rub one temple and sometimes both, according to the effect desired. There is no inconvenience from the application. The surface turns slightly red and feels a little sore upon being touched for two or three days. There is a slight desquamation of the epidermis, and soon there is no sign left of the application.

